# Alcohol consumption habits and their impact on academic performance: analysis of ethanol patterns among health students. A cross-sectional study

**DOI:** 10.1590/1516-3180.2023.0410.R1.05062024

**Published:** 2024-10-21

**Authors:** Ana Paula Amaral de Brito, Aísa de Santana Lima, Átina Carneiro Rocha, Beatriz Muniz Gonçalves, Dalila Maria Costa Baraúna de Freitas, Gleice de Jesus Oliveira, Jamily Kaliny Azevedo Lima, Katia de Miranda Avena

**Affiliations:** I Professor, Departamento de Ciências da Vida, Universidade do Estado da Bahia (UNEB), Salvador (BA), Brazil; Professor, Estrutura e Função II, Medicina Zarns, Salvador, Bahia, Brazil.; II Escola de Ciências da Saúde, Universidade Salvador (UNIFACS), Salvador (BA), Brazil.; III Escola de Ciências da Saúde, Universidade Salvador (UNIFACS), Salvador (BA), Brazil.; IV Escola de Ciências da Saúde, Universidade Salvador (UNIFACS), Salvador (BA), Brazil.; V Escola de Ciências da Saúde, Universidade Salvador (UNIFACS), Salvador (BA), Brazil.; VI Escola de Ciências da Saúde, Universidade Salvador (UNIFACS), Salvador (BA), Brazil.; VII Escola de Ciências da Saúde, Universidade Salvador (UNIFACS), Salvador (BA), Brazil.; VIII Professor Research Supervisor, Medicina Zarns, Salvador (BA), Brazil.

**Keywords:** Alcohol drinking., Binge drinking., Alcohol drinking in college., Alcoholism., Students, health occupations., Drinking Behavior., Epidemiology., Global health., Risk Assessment., Risk Factors., Student Drinking.

## Abstract

**BACKGROUND:**

Studies have indicated a substantial increase in alcohol consumption among university students. Specifically, abusive consumption among health students can adversely affect their academic training and future professional practice.

**OBJECTIVE:**

This study aimed to analyze alcohol consumption habits among healthcare students and investigate the associations between alcohol consumption patterns and sociodemographic and academic variables.

**DESIGN AND SETTING:**

We performed this cross-sectional study at a private university located in the city of Salvador, Bahia.

**METHODS:**

We conducted this study with 770 students using a printed, self-administered, anonymous questionnaire containing sociodemographic and academic performance data, as well as the Alcohol Use Disorders Identification Test (AUDIT) and Rutgers Alcohol Problems Inventory (RAPI).

**RESULTS:**

We observed that the prevalence of alcohol consumption (65.1%) and binge drinking (57.5%) among Brazilian healthcare students was high, with more frequent consumption among men (73.1%), in medicine (83.0%) and veterinary medicine (79.1%) programs and in semesters beyond the fourth (71.7%). We found associations between drinking habits and sex (P = 0.016), religion (P < 0.000), course (P < 0.000) and semester (P = 0.047). Binge drinking was associated with attending academic activities without getting any sleep (P < 0.000), missing classes due to hangovers (P < 0.000), encountering issues with the institution’s administration (P = 0.028), and failing to complete activities due to alcohol consumption (P < 0.000).

**CONCLUSION:**

The prevalence of alcohol consumption and binge drinking among Brazilian healthcare students was high and associated with sex, religion, course, academic semester, risky behaviors, and negative academic impacts.

## INTRODUCTION

Alcohol is the most widely used psychoactive drug globally despite economic, social, and cultural differences among countries worldwide.^1^ Furthermore, it is the substance of choice for consumption among children and adolescents.^2^ It is estimated that three million deaths occur annually worldwide as a result of the harmful use of alcohol, which account for 5.3% of global mortality.^1,2^

In Brazil, alcohol is the most consumed substance across all age groups, and its consumption is increasing among young people.^3,4^ Estimates indicate that Brazilians’ first experience with alcohol begins at the age of 12.^1,2,3,4^ According to the Brazilian Center for Information on Psychotropic Drugs, approximately 20.5% of young people aged 18–24 years face alcohol dependency issues.^5^ These statistics classify this age group as the second most affected, trailing only behind the 25–34-year-old age group, where the dependency rate reaches 23.3%.^5^

Specifically, alcohol consumption in young university students has significantly increased.^6^ It is important to note that university students experience a critical and vulnerable phase in their lives, which may facilitate initial contact and persistence in the consumption of alcoholic beverages. Factors such as leaving the parents’ homes to live alone or with friends and facing new social interactions and experiences can contribute to this scenario.^6^

University life brings about changes in lifestyle habits that may be associated with increased stress levels, more frequent socialization at parties, and exposure to alcoholic beverages.^6,7^ Furthermore, the combination of these social and emotional factors can increase young university students’ susceptibility to occasional abusive alcohol consumption.

It is essential to emphasize that the abusive consumption of alcohol among students in the healthcare field can adversely affect not only their academic education but also their future professional practice, having an impact on both their own health and diagnostic and therapeutic skills.^6^ Considering the inherent vulnerability of university students, identifying their alcohol consumption patterns is important to support the development of preventive interventions that align with harm reduction policies.^7^

## OBJECTIVE

This study aimed to analyze alcohol consumption habits among healthcare students by investigating the associations between alcohol consumption patterns and sociodemographic and academic variables.

## METHODS

We conducted a cross-sectional study with healthcare undergraduate students from a private university located in the city of Salvador, Bahia, from October 2015 to May 2016.

We included students from the Biomedical, Nursing, Pharmacy, Medicine, Veterinary Medicine, Nutrition, Psychology, and Social Work programs starting from the second semester onward. The non-inclusion criterion was being in the first semester of the program owing to the absence of a weighted average, which is a variable of interest in this study. We only included students over 18 years of age.

Sample selection was convenience-based and conducted through voluntary participation, requiring the signing of an informed consent form. We approached potential participants in person on university premises during class breaks and in the morning, afternoon, and evening shifts.

We utilized a printed, self-administered, anonymous questionnaire consisting of three parts. The first part assessed sociodemographic data (sex, age, marital status, and religion) and academic performance (semester-averaged weighted grade, frequency of attending or missing classes after alcohol intake, decrease in academic performance, and noncompletion of academic activities after alcohol intake).

In the second part, we administered the Test for the Identification of Alcohol Problems (AUDIT).^8,9^ Developed by the World Health Organization, the test comprises 10 items aimed at screening alcohol use in the past 12 months, allowing the identification of risky use, harmful use, and alcohol dependence. The AUDIT consists of three questions measuring the quantity and frequency of regular or occasional alcohol use, another three investigating symptoms of dependence, and four related to harmful alcohol use. A score is generated for each of these questions, and risk levels are classified based on this score. Scores range from 0 to 40 points, with scores above 8 indicating the need for a more specific diagnosis.^8^ The sum of the scores defines risk levels as follows: low-risk or abstainers (0–7 points), risky use (8–15 points), harmful use or high-risk use (16–19 points), and probable dependence (20–40 points).

The third part of the questionnaire included an inventory of problems and negative consequences related to alcohol and dependence (the Rutgers Alcohol Problems Inventory [RAPI]).^10^ The RAPI indicates negative behaviors associated with alcohol use, revealing the impact on social functioning and health in the past 12 months and the last month. The instrument consists of 23 items, which are answered as 0 (*never*), 1 (*once or twice*), 2 (*3–5 times*), 3 (*6–10 times*), or 4 (*more than 10 times*). These responses represent the number of times a behavior occurred due to alcohol consumption. The sum of the scores generates a score ranging from 0 to 23 points.

For the analyses, we categorized the participants as drinkers or nondrinkers. Those who answered affirmatively to question 1 of the AUDIT^10^ (“How often do you consume alcoholic beverages?”) were considered drinkers. Subsequently, we classified drinkers as practitioners or nonpractitioners of binge drinking (a pattern of excessive alcohol consumption in a short period).^11,12^ This was determined by considering the consumption of five or more alcoholic drinks (beer, wine, and/or spirits) for men or four or more for women on a single occasion.^13^ Finally, we conducted a descriptive analysis of the study group according to the variables under investigation (sociodemographic variables, academic performance, and risky situations or undesirable events after alcohol use). Additionally, we considered risk behavior when students had an AUDIT score ≥ 8 and/or RAPI score ≥ 7 at any stage in the last 12 months or in the last month.^14^

We initially tabulated the data in Microsoft Office Excel (version 2010, Redmond, Washington, United States) and analyzed them in R software (version 4.0.2, Boston, Massachusetts, United States). We presented qualitative variables as absolute and relative frequencies. To investigate the association between outcome variables (drinking or not drinking /binge drinking or not binge drinking) and potential exposures (presence or absence of each variable) in the group, we performed the chi-square test or Fisher’s exact test, with variables included in the model having a P value < 0.05.

The present study was performed under Resolutions 466/12 and 510/16 of the Brazilian National Health Council and approved by the Brazilian Research Ethics Committee (CAAE: 45396315.8.0000.5033, on September 17, 2015). The participants’ autonomy, confidentiality, and privacy were respected. All study participants were informed of the research objectives and methods and signed an informed consent form.

## RESULTS

A total of 770 students from the healthcare field at a private Brazilian university participated in the study. Among these, most were women (79.2%), aged between 18 and 25 years (82.2%), Catholic (36.0%), residing with family members (83.1%), and in their first two years of undergraduate studies (78.9%) (**Table 1**).

**Table 1:** Sociodemographic and academic characteristics of participating students, considering both the total sample and the grouping based on alcohol consumption (n = 770)

PROFILE*	PARTICIPANTS			P§
	TOTAL (n = 770)	ALCOHOL CONSUMPTION		
		YES (n = 501)	NO (n = 269)	
**Sex**				**0.016**
Men	160 (20.8)	117 (73.1)	43 (26.9)	
Women	610 (79.2)	384 (63.0)	226 (37.0)	
Age**				0.583
18–25 years old	544 (82.2)	355 (65.3)	189 (34.7)	
26–30 years old	63 (9.5)	40 (63.5)	23 (36.5)	
Over 30 years old	55 (8.3)	32 (58.2)	23 (41.8)	
Religion**				**<0.000**
No religion	104 (25.8)	83 (79.8)	21 (20.2)	
Catholic	145 (36.0)	104 (71.7)	41 (28.3)	
Protestant	92 (22.8)	31 (33.7)	61 (66.3)	
Spiritist	37 (9.2)	29 (33.7)	8 (21.6)	
African religions	3 (0.7)	3 (100.0)	0 (0.0)	
Eastern religions	1 (0.2)	1 (100.0)	0 (0.0)	
Others	21 (5.2)	10 (47.6)	11 (52.4)	
Residence**				0.093
With Family	343 (83.1)	227 (66.2)	116 (33.8)	
With Friends	29 (7.0)	24 (82.8)	5 (17.2)	
Alone	16 (3.9)	14 (87.5)	2 (12.5)	
Others	25 (6.1)	18 (72.0)	7 (28.0)	
**Programs**				**<0.000**
Biomedical	131 (17.0)	56 (42.7%)	75 (57.3%)	
Medicine	100 (13.0)	83 (83.0%)	17 (17.0%)	
Nursing	143 (18.6)	85 (59.4%)	58 (40.6%)	
Nutrition	129 (16.8)	96 (74.4%)	33 (25.6%)	
Pharmacy	19 (2.5)	1 (5.3%)	18 (94.7%)	
Psychology	26 (3.4)	17 (65.4%)	9 (34.6%)	
Social Work	136 (17.7)	95 (69.9%)	41 (30.1%)	
Veterinary Medicine	86 (11.0)	68 (79.1%)	18 (20.9%)	
Academic semester in progress**				**0.047**
≤ 4th	593 (78.9)	375 (63.2)	218 (36.8)	
> 4th	159 (21.1)	114 (71.7)	45 (28.3)	

*Data presented as absolute frequency (n) and relative frequency (%);

**Relative frequency calculated considering the number of responses obtained for each variable (age: n = 662; religion: n = 403; residence: n = 413; semester: n = 752);

^§^Chi-squared test or Fisher’s Exact test.

We observed that 65.1% of the interviewed students used alcoholic beverages, with this prevalence being high among both men (73.1%) and women (63.0%). Furthermore, alcohol consumption was more frequent among students in semesters beyond the fourth (71.7%), with notable rates in the fields of medicine (83.0%), veterinary medicine (79.1%), and nutrition (74.4%) (**Table 1**).

An analysis of the associations between sociodemographic and academic data and alcohol consumption revealed that sex (P = 0.016), religion (P < 0.000), course (P < 0.000), and semester (P = 0.047) were associated with drinking habits. Conversely, age and living situation were not significantly associated with such habits (**Table 1**).

Among the students who reported consuming alcoholic beverages, most exhibited low consumption levels; we classified these individuals as low-risk drinkers (64.9%) (**Table 2**).

**Table 2:** Alcohol consumption categorized using the Alcohol Use Disorders Identification Test (AUDIT)

ALCOHOL CONSUMPTION CLASSIFICATION	PARTICIPANTS* (n = 501)
Low Consumption	325 (64.9)
Risky Behavior	148 (29.5)
High Risk or Harmful Use	6 (1.2)
Dependence	22 (4.4)

* Data presented as absolute frequency (n) and relative frequency (%).

We observed binge drinking in 57.5% of the participants who reported consuming alcoholic beverages and identified risky behavior in 63.9% of binge drinkers and 14.6% of non-binge drinkers. We observed a significant association between this practice and the adoption of risky behavior (P < 0.000) (**Table 3**).

**Table 3:** Association between risky behavior and the practice of binge drinking among participating students who reported alcohol consumption (n = 501)

RISKY BEHAVIOR*	BINGE DRINKING**		P§
	YES (n = 288)	NO (n = 213)	
Presence	184 (63.9)	31 (14.6)	**< 0.000**
Absence	104 (36.1)	182 (85.4)	

*Risky behavior was considered when AUDIT ≥ 8 and/or RAPI ≥ 7 at any phase, in the last 12 months, or in the last month;

** Data presented as absolute frequency (n) and relative frequency (%);

** Chi-square test of independence.

When analyzing the sociodemographic and academic profiles of students who reported consuming alcoholic beverages and engaging in binge drinking, we found a significant association with the variable of religion (P = 0.002) but no associations between binge drinking and the other variables studied (**Table 4**).

**Table 4:** Association between the sociodemographic and academic profile of students who reported alcohol consumption and the practice of binge drinking (n = 501)

PROFILE*	BINGE DRINKING*		P**
	YES	NO	
**Sex**			0.710
Men	69 (59.0)	48 (41.0)	
Women	219 (57.0)	165 (43.0)	
**Age**			0.238
18–25 years old	202 (56.9)	153 (43.1)	
26–30 years old	21 (52.5)	19 (47.5)	
Over 30 years old	15 (46.9)	17 (53.1)	
**Religion**			**0.002**
No religion	39 (47.0)	44 (53.0)	
Catholic	65 (62.5)	39 (37.5)	
Protestant	11 (35.5)	20 (64.5)	
Spiritist	16 (55.2)	13 (44.8)	
African religions	0 (0)	3 (100.0)	
Eastern religions	0 (0)	1 (100.0)	
Others	9 (90.0)	1 (10.0)	
**Residence**			0.392
With Family	125 (55.1)	102 (44.9)	
With Friends	16 (66.7)	8 (33.3)	
Alone	6 (42.9)	8 (57.1)	
Others	12 (66.7)	6 (33.3)	
**Programs**			0.113
Biomedical	28 (50.0)	28 (50.0)	
Medicine	52 (62.7)	31 (37.3)	
Nursing	47 (55.3)	38 (44.7)	
Nutrition	47 (49.0)	49 (51.0)	
Pharmacy	0 (0)	1 (100.0)	
Psychology	11 (64.7)	6 (35.3)	
Social Work	65 (68.4)	30 (31.6)	
Veterinary Medicine	38 (55.9)	30 (44.1)	
**Academic semester in progress**			0.066
≤ 4th	224 (59.7)	151 (40.3)	
> 4th	57 (50.0)	57 (50.0)	

*Data presented as absolute frequency (n) and relative frequency (%);

** Chi-square test of independence.

When assessing the effects of alcohol consumption on academic life, we found that binge drinking was significantly associated with attending academic activities without getting any sleep (P < 0.000), missing classes due to hangovers (P < 0.000), encountering issues with the institution’s administration (P = 0.028), and failing to complete activities due to alcohol consumption (P < 0.000). However, we observed no associations with attending the institution while intoxicated, achieving weighted averages less than 7.0 points in the last semester, or experiencing a decline in academic performance in the last semester (**Table 5**).

**Table 5:** Association between binge drinking and the effects of alcohol consumption on the academic life of participating students

QUESTIONS	BINGE DRINKING*		P**
	YES	NO	
**Have you ever attended academic activities without getting any sleep?**			**< 0.000**
Yes	88 (80.7)	23 (50.0)	
No	21 (19.3)	23 (50.0)	
**Have you ever skipped class due to a hangover?**			**< 0.000**
Yes	68 (23.9)	24 (11.4)	
No	216 (76.1)	186 (88.6)	
**Have you ever attended the educational institution while drunk?**			0.195
Yes	33 (11.5)	17 (8.0)	
No	254 (88.5)	196 (92.0)	
**Have you ever experienced issues with the school administration due to negative behaviors resulting from excessive drinking?**			**0.028**
Yes	10 (3.5)	1 (0.5)	
No	274 (96.5)	212 (99.5)	
**Have you ever failed to fulfill your responsibilities due to drinking?**			**< 0.000**
Yes	44 (15.3)	11 (5.2)	
No	243 (84.7)	201 (94.8)	
**Is your weighted average grade from the last semester less than 7 points?**			0.141
Yes	11 (3.9)	14 (6.9)	
No	271 (96.1)	189 (93.1)	
**Have you experienced a decrease in academic performance in the last semester?**			0.443
Yes	23 (8.6)	13 (6.7)	
No	243 (91.4)	181 (93.3)	

*Data presented as absolute frequency (n) and relative frequency (%);

** Chi-square test of independence.

## DISCUSSION

When investigating the alcohol habits of students, the present study demonstrated a substantial rate of alcohol consumption among Brazilian healthcare students, with relevant associations between sex, religion, course, academic semester, and drinking habits. Moreover, a considerable number of students disclosed their involvement in binge drinking—a behavior linked to high-risk conduct and adverse effects on academic achievement.

Studies conducted by several universities around the world have corroborated the prevalence of alcohol consumption shown in the present study, indicating that the frequency of alcohol intake among healthcare students ranges from 57.5%^6^ to 85.0%.^12^ These results are higher than the average for the general population, which is 40%.^1^ Furthermore, in addition to demonstrating a prevalence of 81.6% alcohol consumption among university students, Pedrosa et al.^15^ showed that alcohol consumption tends to increase over the years. It is important to note that while the onset of this behavior may precede university entrance, the academic environment can contribute to intensifying the habit of drinking.^16^

Regarding the profile of alcohol consumers, we observed a predominance of this practice among men, which is consistent with the findings of other studies.^5,17,18^ Additionally, data from the Third National Survey on Drug Use by the Brazilian Population^5^ indicate that dependence is 3.4 times more common in men than in women. This fact was also highlighted in the study by Silva et al.,^18^ which, in addition to demonstrating a higher prevalence in men, showed a growing prevalence of abusive alcohol consumption in the Brazilian adult population. Specifically regarding the city of Salvador, recent data from the Vigitel 2023 survey^19^ indicate that the capital of Bahia leads the country in alcohol abuse consumption, both for the general population and for men and women, surpassing national rates. In Brazil, the prevalence of alcohol abuse consumption in the general population is 20.8%, while in Salvador, it is 28.9%.^19^ Given the high rate of alcohol abuse consumption in this population, this pattern of behavior may also be observed among the student participants in the study.

Although changes in social patterns favor behavioral convergence between sexes, resulting in increased alcohol intake among women over the years, it remains evident that a male predominance exists among alcohol consumers. According to Fachini et al.,^20^ the intensity with which men drink is associated with positive cultural perceptions linked to use, such as the possibility of improved sexual performance.

Although the current study did not reveal substantial differences in alcohol consumption based on age, prior research has suggested a slight prevalence of alcohol consumption among university students aged 21–25 years.^21^ Similarly, the housing variable did not significantly differ; however, we noted a trend toward a considerable percentage of alcohol consumption among students who lived alone or with friends. These findings corroborate studies indicating that students residing apart from family members often tend to exhibit elevated levels of alcohol consumption, which is potentially attributed to reduced parental oversight over their behavior.^22^

Regarding the course attended by university students, medical students have reported higher alcohol consumption, which aligns with the findings of Gomes et al.^23^ According to Abreu et al.,^21^ the pronounced susceptibility of this population to alcohol consumption may be associated with the characteristics of the course that predisposes individuals to stressful situations, such as a high workload combined with a large amount of content to study, excessive demands both inside and outside the academic environment, and delayed financial independence.

Considering that these young medical students will be future leaders in actions related to the prevention, diagnosis, and treatment of disorders associated with abusive alcohol consumption, it is concerning to find that this behavior can transcend graduation and potentially interfere with the quality of their professional practice. This problem is not limited to Brazilian students; a study conducted in the United States revealed that 12.9% of male medical students and 21.4% of female medical students met the criteria for alcohol abuse or dependence.^24^

Regarding the relationship between alcohol consumption and academic semester, the present study revealed that alcohol consumption was more frequent among students enrolled from the fifth semester onward. A similar finding was reported by Gomes et al.,^25^ who demonstrated a progressive increase in alcohol consumption among medical students of both sexes over semesters, reaching 93.6% in the fifth semester.

Although the literature does not specify a period of greater vulnerability to drug use within the university, certain situations in the educational context, such as parties and stressful course conditions, vary throughout the semester and may provide greater access to alcoholic beverages.^22,26^ Additionally, as they advance in their courses, healthcare students, who naturally acquire greater knowledge about the topic, may develop the conviction that they can manage any problems that may arise due to improper alcohol and drug use.^21^

In the present study, when analyzing the risk zones established by the AUDIT, more than half of the sample was classified in the low-risk zone, as in the study by Imai et al.^17^ Furthermore, a minority of the study population fell into the categories of harmful use and probable dependence, which echoed the findings of Imai et al.^17^ and Barbosa et al.^22^

In this context, the abusive and increasing consumption of psychoactive substances—especially alcohol—among young university students represents a considerable challenge for public health due to the interference of alcohol as a cause of disease, morbidity, and mortality worldwide.^27^ Among university students, healthcare students are among the most vulnerable to binge drinking—a phenomenon characterized by consuming high doses of alcohol (four or more drinks for women and five or more for men) on a single occasion.^11,21,28^

The physiopathological, social, and economic impacts resulting from abusive alcohol consumption are widely documented. However, a better understanding of the underlying reasons that make healthcare students particularly vulnerable to this behavior is still necessary. The high rate of consumption and abuse in the academic environment may reflect the ease of accessing alcoholic beverages at frequent university parties and the adaptation mechanism to the academic environment, which ranges from its use as a means of social inclusion to a refuge from the stressful factors associated with courses.^12^

In the present study, a substantial portion of students engaged in binge drinking. Compared with other similar Brazilian studies, the prevalence of binge drinking varies widely, ranging from 15.6%^29^ to 56.1%^12^; this suggests variability among regions and student populations, emphasizing the importance of specific prevention approaches for each situation.

Another relevant point is that heavy drinking habits can interfere with the development of these future professionals. According to a study by Cardoso et al.,^30^ this habit can contribute to unpreparedness in providing healthcare. Thus, this unpreparedness in the profession results from the interference of abusive use in essential work activities, such as learning, memory, psychomotor speed, and decision-making.^21^

We observed an association between religion and both drinking habits and binge drinking practices. University students who declared themselves Protestants had the lowest alcohol consumption, while those who were Catholic reported the highest consumption. Individuals without religious ties have a high prevalence of abusive alcohol use^29^; this characterizes religion as a protective factor against the adoption of risky behaviors, including the abusive use of substances such as alcohol. The fact that religiosity establishes norms and values that directly and indirectly influence individual attitudes may favor the rejection of drug use.

However, religions show differences with regard to the values that modulate the acceptance of consumption, which may be more or less permissive, especially about alcohol use. Therefore, Silva et al.^31^ have concluded that religions with less permissive views on alcohol consumption, such as Protestantism, played a more determining role in preventing abusive use.

The present study demonstrated the negative effects of binge drinking on academics through practices such as attending academic activities while intoxicated, missing class due to hangovers, experiencing administrative issues with the educational institution, and not completing academic activities due to alcohol consumption. The impact of binge drinking on the academic environment, whether through absence from activities or impaired performance, was also identified in the study by Cardoso et al.^30^ Furthermore, binge drinking practitioners can be considered to exhibit harmful behavior to individual and collective health. The concept of harmful behavior encompasses everything from dependence on health hazards and chronic diseases^32^ as well as actions that directly affect society, such as legal problems and involvement in violent actions secondary to abusive use.^27^ The importance of the topic is supported by the various negative effects of abusive use and its impact on public health, which significantly contribute to morbidity and mortality.

Given these findings, this study is expected to inform future research and facilitate the creation of university-based prevention policies that aim to mitigate the adverse effects of substance abuse, enhance students’ quality of life, and support their professional development. This includes the implementation of preventive measures in educational institutions that disseminate information about the consequences of abusive consumption, as well as the identification of at-risk groups, so that therapeutic interventions can be offered.^22^

Finally, the limitations of this study include the low participation of students in semesters with few in-person classes at the institution due to more practical fieldwork, which limits the application of the data-collection instrument. As the questionnaire is self-administered and extensive, one must consider possible comprehension difficulties and fatigue in responding to all its questions. These factors may have contributed to some questions not being answered in their entirety by all participants in the sample. Despite these potential limitations, the findings presented in this study can be considered relevant to the scientific literature.

## CONCLUSION

This study revealed high prevalence rates of alcohol consumption and binge drinking among healthcare students at a private university, with associations between these patterns of consumption and sex, religion, course, and academic semester. Additionally, these habits were associated with risky behaviors and negative impacts on academic performance due to noncompliance with curricular activities and the development of administrative problems with educational institutions.
